# Weak or no association of *TCF7L2 *variants with Type 2 diabetes risk in an Arab population

**DOI:** 10.1186/1471-2350-9-72

**Published:** 2008-07-26

**Authors:** Osama Alsmadi, Khalid Al-Rubeaan, Gamal Mohamed, Fadi Alkayal, Haya Al-Saud, Nouran Abu Al-Saud, Nasser Al-Daghri, Shahinaz Mohammad, Brian F Meyer

**Affiliations:** 1 Saudi Diagnostic Laboratory (SDL), Research Centre, King Faisal Specialist Hopital and Research Center, Riyadh, Kingdom of Saudi Arabia; 2 Diabetes Center, King Abdulaziz University Hospital, Riyadh, Kingdom of Saudi Arabia; 3 Department of Biostastics, Epidemiology and Scientifc Computing, King Faisal Specialist Hospital and Research Centre, Riyadh, Kingdom of Saudi Arabia

## Abstract

**Background:**

The rs7903146 and rs12255372 variants of *TCF7L2 *have been strongly associated with type 2 diabetes (T2D) risk in most populations studied to date. Meta-analysis of 27 different studies has resulted in a global OR of 1.46 [1.42–1.51] (rs7903146 variant). Thus far, despite a high incidence of T2D, the role of this variant in Arabs has not been established.

**Methods:**

We performed a case-control association study using 522 Saudi T2D patients (WHO criteria), and 346 controls (age > 60; fasting plasma glucose < 7 mmol/L). Genotyping was performed by pyrosequencing. Statistical analyses were performed using SPSS version 13.0 for Windows (SPSS, Chicago, IL, USA).

**Results:**

For rs7903146, the T allele frequency of the cases (0.415) was not different from that observed in the controls (0.405). The crude odds ratio (OR) was 1.04 with a 95% CI of 0.86–1.27 (P = 0.675). For rs12255372, the T allele frequency of the cases (0.368) was not different from that observed in the controls (0.355). Retrospective power calculations based upon an OR of 1.46 reported in a comprehensive meta-analysis of *TCF7L2 *risk, indicated this study was sufficiently powered (96.92%; α = 0.05) to detect an effect of similar magnitude to that reported for rs7903146.

**Conclusion:**

Our study is consistent with weak or no association of T2D in Arabs with the two *TCF7L2 *variants, however it cannot rule out an effect of other SNPs in this gene. Future studies in this population are required to confirm our findings and may indicate the presence of yet to be defined genetic risk factors for T2D.

## Background

Type 2 diabetes (T2D) is one of the most common non infectious diseases globally and is a health-care problem worldwide affecting both industrial and developing nations [1]. A clear link has been made between genetic defects and the less common monogenic forms of diabetes, such as maturity onset diabetes of the young (MODY) and neonatal diabetes [2,3]. Conversely, the genetics underlying type 2 diabetes is multifactorial and complex in nature[4]. Multiple genetic and environmental factors contribute to individual risk of developing type 2 diabetes. Some have only a marginal and modest effect when considered individually, and the combination of these factors is responsible for disease risk. Most genetic studies that have addressed these factors have not provided reproducible conclusions [5]. Studies which investigated the P12A variant in *PPARG *and E23K in *KCNJ11 *have provided only a modest disease risk and hence presented little explanation to the disease etiology [6,7]. More recently, researchers at Decode Genetics reported strong association between variants in a novel susceptibility gene called *TCF7L2 *and type 2 diabetes in Icelandic diabetic patients [8]. *TCF7L2 *encodes the transcription factor 7-like 2 [9]. The overexpression of this gene in human pancreatic β cells was shown to associate with impaired insulin secretion both in vivo and in vitro [10]. This gene received attention from many research groups following this report, and similar studies were replicated in samples from several populations. Many studies have confirmed the original findings. Substantial association has been confirmed between variants in *TCF7L2 *and type 2 diabetes among broad ethnic backgrounds, including for example populations of UK [11], Dutch [12], Amish [13], Finnish [14], Swedish [15], French [16] and US [17,18], Indian [19], and Japanese [20] origin. It is noteworthy that, as in the original report, there was clear evidence of a gene dosage effect, such that the 10% of individuals with two copies of the susceptibility allele were at almost twice the risk of developing type 2 diabetes compared to those with only one copy [11,21]. Very recently, lack of association between variants in *TCF7L2 *and type 2 diabetes has been reported in Pima Indians and Chinese diabetics [22,23]. In another association study performed in Emirati Arabs [24], the authors reported only a marginal association between rs12255372 and type 2 diabetes risk and no association with rs7903146. We have previously studied the P12A [25] and E23K [26] variants in a cohort of T2D subjects of Arabian origin. Our aim in this study was to replicate the *TCF7L2 *gene studies in these same subjects. We focused on two of the best studied SNPs in this gene (rs7903146 and rs12255372) to examine whether they contribute to the risk of type 2 diabetes in an Arab population using unrelated subjects and non-diabetic controls of Saudi origin.

## Research design and methods

### Subjects

Random unrelated Saudi T2D patients (522) were recruited through a program for the Genetic Study of Saudi Diabetes (GSSD). Diagnosis was based upon WHO criteria (fasting plasma glucose > 7.0 mmol/l, and/or 2 hr OGTT ≥ 11.1). Their age ranged between 60 and 88 years. Control subjects (346) were random unrelated anonymised individuals, aged between 60–95 years with a fasting plasma glucose < 7.0 mmol/l (highly unlikely to develop T2D). Patient's participation in this study was with full informed consent based on the principles of the Declaration of Helsinki and as required by the Institutional Review Boards of King Faisal Specialist Hospital and Research Center and The University Diabetes Center at King Saud University.

### Blood sample collection and DNA extraction

Peripheral blood was drawn from type 2 diabetes patients and controls in EDTA anticoagulated tubes. DNA was extracted from whole blood using the PUREGENE DNA Isolation Kit (Gentra Systems, Minneapolis, MN, USA) as recommended by the manufacturer. DNA was quantified spectrophotometrically prior to use in PCR.

### PCR and pyrosequencing

PCR reaction consisted of 2.0 μL Qiagen 10× Buffer containing 15 mM MgCl2 solution (Qiagen, Valencia, CA, USA), 2 μL of 2.5 mM dNTP (EPICENTER Biotechnologies, Madison, WI, USA), 1 μL of 5 pmol/μL forward primer and 1 μL of 5 pmol/μL 5' BIOTINYLATED reverse primer (Metabion, Germany), 2 μL of 25 ng/μL DNA template, 0.2 μL (1 unit) Taq polymerase (Qiagen, Valencia, CA, USA), and sterile water to 20 μL total reaction volume. PCR thermal cycling was done on PTC-200 (MJ Research, Watertown, MA, USA) using the following conditions: "heated lead", 95°C for 15 min; 35 cycles consisting of denaturation at 95°C for 30 s, annealing at 54°C for 30 s, and extension at 72°C for 30 sec; and final extension at 72°C for 5 min. PCR primers were designed using PSQ Assay Design Software (PyroMark™ MD version 1.0, Biotage, Sweden). Pyrosequencing was performed for sequence determination and allele designation in a Biotage PSQ HS 96 System, and data were captured with PSQ HS 96 SNP software. Pyrosequencing was performed as described previously [27]. Briefly, the biotinylated PCR products (20 μL) were immobilized onto streptavidin-coated Sepharose Dynabeads™ M280-streptavidin (Dynal A.S., Oslo, Norway). Using Pyrosequencing wash unit, Single-stranded DNA sequencing template was obtained by discarding the supernatant after incubation of the bead-immobilized PCR biotinylated strand in 0.10 M NaOH for 30 sec. Beads were then treated with 70% ethanol, denaturation buffer, wash buffer, and released into a 96 well PSQ sequencing plate containing annealing buffer and 10 pmol of the appropriate sequencing primer. The plate was heated at 80°C for 5 min, cooled to 55°C for 4 min, and then allowed to stay at room temperature. Primed DNA was finally placed in the PSQ™ Pyrosequencing machine. Pyrosequencing™ substrate and enzymes were dispensed using the fully automated microtiter plate-based Pyrosequencing machine. The progress of sequencing was followed in real time using Pyrosequencing SNP software . Genotypes for 96-well plates were generated in 10 min. For quality control, replicate 96-well plates (one full plate per each SNP) were included in the analysis and genotyped with 100% concordance. SNP genotyping success rates also ranged between 93–98% for the successive runs.

### Statistical analysis

Genotype frequencies were tested for Hardy-Weinberg equilibrium by χ^2 ^analysis. Mean age of cases and controls was compared by t-test. Multiple logistic regression was used to study the effect of each allele on the risk of diabetes when adjusting for age and sex. Differences in case and control groups for allele, genotype and haplotype frequencies were tested for significance using a 95% two-sided χ^2 ^test. Individuals homozygous for the common allele were used as reference, to test for association of genotype with T2D using a logistic regression model to calculate Odds ratios (ORs) with 95% confidence intervals (CIs). Haplotypes of the two SNPs were estimated using the SAS Genetics statistical software package (SAS Institute Inc., Cary, NC, USA, 2002). Estimates of linkage disequilibrium (LD; *D' *and *r*^2^) between rs7903146 and rs12255372 were made using the same package. All statistical analyses were performed using SPSS version 13.0 for Windows (SPSS, Chicago, IL, USA) unless otherwise indicated.

## Results

We conducted a case-control association study comprising 522 T2D patients and 346 controls, both aged above 60 years (Table 1). Both groups were of Saudi ancestry and representative of an Arab population. The rationale behind the minimum age selection was to ensure the absence of silent diabetics within the control group. The male gender distribution was 58.1% (201) vs 57.5% (300) among controls and patients respectively, there being no significant difference in the groups, *p *= 0.856. The age range of controls and cases respectively was 60–95 and 60–88 years. The mean age was 68.6 (± 7.0) and 67.2 (± 6.0) for controls and patients respectively and was found to be significantly different (p = 0.002) (Table 1).

**Table 1:** Description of study controls and type 2 diabetes subjects

Subjects	Controls (n = 346)	Diabetics (n = 522)	p-value
BMI (kg/m^2^)	NA	28.9 ± 5.48	-
Male distribution (%)	58.1 (201/346)	57.7 (300/522)	0.856
Mean age (years)	68.6 ± 7.0	67.2 ± 6.0	0.002
Age range (years)	60–95	60–88	-

For rs7903146, the T allele frequency of the cases (0.415) was not different from that observed in the controls (0.405) (Table 2). The crude odds ratio (OR) was 1.04 with a 95% CI of 0.86–1.27 (P = 0.675) (Table 2). Retrospective power calculations based upon an OR of 1.46 reported in a comprehensive meta-analysis of *TCF7L2 *risk [28], indicated this study was sufficiently powered (96.92%; α = 0.05) to detect an effect of this magnitude or greater. The CT genotype frequency of the cases was 0.485 compared to 0.468 among the controls, the difference was not significant when compared to the CC reference genotype (OR 1.09, 95% CI 0.81–1.47, P = 0.573). TT genotype frequency of both the cases and controls was also compared to the CC reference genotype and resulted in a P value of 0.757, and an OR of 1.065 with a 95% CI of (0.71–1.59). This indicated no significant difference between the cases and controls with respect to CT and TT genotypes (Table 2).

**Table 2:** Univariate analysis relating allele and genotype to the risk of diabetes

Subjects	Controls	Diabetics	Odds ratio (95% CI)	p-value
SNP genotypes				

**rs7903146**				

C allele (%)	412 (59.5)	611(58.5)		-
T allele (%)	280(40.5)	433(41.5)	1.04 (0.86–1.27)	0.675
CC genotype (%)	125(36.1)	179(34.3)		-
CT genotype (%)	162(46.8)	253(48.5)	1.09 (0.81–1.47)	0.573
TT genotype (%)	59(17.1)	90(17.2)	1.065 (0.71–1.59)	0.757

**rs12255372**				

G allele (%)	446(64.5)	660(63.2)		
T allele (%)	246(35.5)	384(36.8)	1.06 (0.86–1.29)	0.601
GG genotype (%)	142(41.0)	211(40.4)		
GT genotype (%)	162(46.8)	238(45.6)	0.989 (0.74–1.32)	0.939
TT genotype (%)	42(12.1)	73(14.0)	1.17 (0.76–1.81)	0.48

For rs12255372, the T allele frequency of the cases (0.368) was not different from that observed in the controls (0.355) (Table 2). The crude odds ratio (OR) was 1.06 with a 95% CI of 0.86–1.29 (P = 0.601) (Table 2). The GT genotype frequency of the cases was 0.456 compared to 0.468 among the controls, the difference was not significant when compared to the GG reference genotype (OR 0.989, 95% CI 0.74–1.32, P = 0.938). TT genotype frequency of both the cases and controls was also compared to the GG reference genotype. The statistical analysis resulted in a P value of 0.48, and an OR of 1.17 with a 95% CI of (0.76–1.81). This also indicated no significant difference between the cases and controls with respect to rs12255372 GT and TT genotypes (Table 2).

It was noted that the cases and controls differed slightly with respect to age. Multiple logistic regression analysis was used to correct for the effect of age and sex on genotype (Tables 3 &4). Data showed that, even after adjustment for the joint effects of sex and age, the genotypes were not significantly associated with T2D.

**Table 3:** rs7903146 multiple logistic regression to see the effect of genotype on the risk of diabetes after controlling for age and sex

Variables	Odds ratio	95.0% C.I.for Odds ratio		P-value
			
		Lower	Upper	
Sex (female reference)	.997	.755	1.315	.982
age	.966	.946	.987	.001
rs4903146				.856
CC	Reference			
CT	1.061	.709	1.587	.774
TT	1.090	.804	1.477	.579

**Table 4:** rs12255372 multiple logistic regression to see the effect of genotype on the risk of diabetes after controlling for age and sex

	Odds ratio	95.0% C.I.for Odds ratio		
			
Variable		Lower	Upper	p-value
Sex (female reference)	.992	.751	1.309	.953
Age	.966	.946	.987	.001
rs12255372				.734
GG (reference)				
GT	.993	.740	1.331	.960
TT	1.173	.757	1.819	.474

The allele frequencies for rs7903146 and rs12255372 SNPs in cases and controls were in Hardy-Weinberg equilibrium and both SNPs were found to be in modest LD with each other (*D' *= 0.8, *r2 *= 0.66). The frequency of the TT haplotype, which carries the two minor alleles (risk alleles in other populations) of both SNPs, was not significantly different in subjects compared to controls (33% vs 30% respectively; p = 0.1373). Differences between cases and controls for the other haplotypes of rs7903146 (C/T) and rs12255372 (G/T) were not significantly different with the exception of the CT haplotype (p = 0.0312). However, the CT haplotype was only observed in a combined total of 38 individuals from cases and is underpowered for such comparison.

In order to determine whether the at risk variants of rs7903146 and rs12255372 interacted with body mass index (BMI) we studied the association between these alleles and this covariate in 200 T2D cases from this study for which BMI was available. The mean BMI for this group of patients was 28.9 ± 5.5. The mean BMI for the C and T allele of rs7903146 was 28.7 ± 5.4 and 29.1 ± 5.6 respectively (P = 0.508). Similarly the mean BMI for the G and T alleles of rs12255372 (28.6 ± 5.4 and 29.4 ± 5.7 respectively) were not significantly different (P = 0.190). The association of BMI with genotypes of rs7903146 and rs12255372 was also not statistically significant (P = 0.442 and P = 0.078 respectively).

## Discussion and conclusion

Variants in *TCF7L2 *have been strongly associated with type 2 diabetes risk [28]. In this study, we aimed to explore the effects of rs7903146 and rs12255372 variants in an Arab population. Previous reports [11-14,16-19,29] have suggested a positive association between *TCF7L2 *variants and T2D. In our case-control study, we found that rs12255372 and rs7903146 were not associated with T2D making this the first study to find simultaneously no association between these variants in *TCF7L2 *and T2D in Arabs. Several studies from non-European ethnic backgrounds have reported a positive association between *TCF7L2 *variants and T2D. The first, an Indian study, investigated 3 *TCF7L2 *variants (rs7903146, rs12255372, and rs4506565) and reported significant association between all 3 SNPs and T2D [19]. In a Japanese study, 4 *TCF7L2 *SNPs were explored (rs12255372, rs7903146, rs7901695 and rs11196205) and all 4 SNPs were found to be significantly associated with T2D, with rs12255372 showing the strongest association [20]. The third study was conducted by Cauchi et al. on Moroccans [28]. Significant association between rs7903146 variant of *TCF7L2 *and T2D risk in this population was concluded. Additionally, positive association was also reported on Indian Asians [30,31], Pakistanis [32], and Afro-Caribbeans [31]. More recently, a surprising lack of association between *TCF7L2 *variants and type 2 diabetes was independently reported in three non-European populations including Chinese [22], Pima Indians [23], and in respect to rs7903146 in Emirati Arabs [24]. In a systemic meta analysis conducted by Cauchi et al., the authors reviewed the association of rs7903146 variant with T2D risk by looking at 27 original published association studies (including their own), the authors arrived at a pooled OR of 1.46 [1.42–1.51]. Whilst there is no overlap between the over all OR and CIs of this meta-analysis and the upper CI of our study (1.27), however there is an overlap with three studies included in this meta-analysis [17,31,33]. Therefore, even though significant association was not indicated by our study, a weak association cannot be ruled out and justifies a larger replication study in Arabs. Within T2D patients of this population, BMI was not associated with allele frequencies for rs12255372 and rs7903146 SNPs. This is consistent with studies performed in other populations where BMI was essentially similar to that reported in our study [8,12,17,18].

*TCF7L2 *variants linked to T2D so far are all intronic, and there is no clear mechanism so far linking T2D pathogenicity to this gene. *TCF7L2 *is widely expressed [16], however little is known about its biological role and the predisposition to diabetes. Current evidence suggests the idea that the predominant effect of *TCF7L2 *dysfunction on type 2 diabetes development is mediated through impairment of insulin secretion [13,14,17,19,21]. A meta-analysis of 27 different populations confirmed association of rs7903146 with T2D in diverse populations with a global OR of 1.46 [1.42–1.51] showing a uniform risk conferred through the effect of *TCF7L2*. The absence of heterogeneity among studies was considered indicative of a universal contribution of this gene to T2D [28]. Whilst *TCF7L2 *clearly confers yet the strongest and most widespread association with T2D, our results suggest caution should be exercised, especially in light of other very recent reports [22,23] which have also shown that these two *TCF7L2 *variants are not associated with type 2 diabetes in other ethnic populations. This is supported further by the lack of association between type 2 diabetes and rs7903146 and marginal association with rs12255372 in Emirati Arabs, a population with a similar incidence of type 2 diabetes and associated phenotypes[24]. Whilst marginal association of the T allele of rs12255372 with type 2 diabetes was reported in this study, cohort size was small (180 cases and 188 controls) as evidenced by the broad CI (1.04 – 2.08) for the OR of 1.47. Indeed the CI overlaps considerably with our findings and highlights the need for replication studies in a larger cohort of Arabs. The possibility that other SNPs in *TCF7L2 *may influence the disease risk in these populations cannot be excluded.

## Competing interests

The authors declare they have no competing interests.

## Authors' contributions

OA designed the study, analyzed the data, and wrote the manuscript, KA carried out the clinical assessment and patient phenotyping. GM carried out the statistical analysis. FA, HAS, and NAA conducted the molecular genotyping of the variants. NAD and SM coordinated the patients' recruitment. BFM assisted in the study design and manuscript writing and editing. All authors read and approved the final manuscript.

## Pre-publication history

The pre-publication history for this paper can be accessed here:


